# Exploring chromatin structural roles of non-coding RNAs at imprinted domains

**DOI:** 10.1042/BST20210758

**Published:** 2021-08-02

**Authors:** David Llères, Yui Imaizumi, Robert Feil

**Affiliations:** 1 Institute of Molecular Genetics of Montpellier (IGMM), Centre National de Recherche Scientifique (CNRS), Montpellier, France; 2 University of Montpellier (UM), Montpellier, France

**Keywords:** chromatin, CTCF, epigenetics, genomic imprinting, non-coding RNA

## Abstract

Different classes of non-coding RNA (ncRNA) influence the organization of chromatin. Imprinted gene domains constitute a paradigm for exploring functional long ncRNAs (lncRNAs). Almost all express an lncRNA in a parent-of-origin dependent manner. The mono-allelic expression of these lncRNAs represses close by and distant protein-coding genes, through diverse mechanisms. Some control genes on other chromosomes as well. Interestingly, several imprinted chromosomal domains show a developmentally regulated, chromatin-based mechanism of imprinting with apparent similarities to X-chromosome inactivation. At these domains, the mono-allelic lncRNAs show a relatively stable, focal accumulation in *cis*. This facilitates the recruitment of Polycomb repressive complexes, lysine methyltranferases and other nuclear proteins — in part through direct RNA–protein interactions. Recent chromosome conformation capture and microscopy studies indicate that the focal aggregation of lncRNA and interacting proteins could play an architectural role as well, and correlates with close positioning of target genes. Higher-order chromatin structure is strongly influenced by CTCF/cohesin complexes, whose allelic association patterns and actions may be influenced by lncRNAs as well. Here, we review the gene-repressive roles of imprinted non-coding RNAs, particularly of lncRNAs, and discuss emerging links with chromatin architecture.

## Introduction

Diverse genetic and epigenetic systems of mono-allelic expression have evolved in mammals, together controlling thousands of genes [1]. These mono-allelic gene expression mechanisms provide unique identities to cells, such as in hematopoietic cells or olfactory neurons, or critically modulate the dosage of gene expression, such as in X-chromosome inactivation in females [1]. The epigenetic phenomenon of genomic imprinting is exceptional in that this kind of mono-allelic expression depends entirely on the parental origin of the gene [2]. Some imprinted genes are expressed from the maternally inherited copy only, others only from the paternal copy. About 150 genes are known to be imprinted in humans and mice [3,4] and their correct expression levels are important for fetal growth, development, homeostasis and behavior [5,6].

Imprinting is controlled by oocyte- and sperm-derived DNA methylation marks put onto specialized CpG islands called ‘imprinting control regions’ (ICRs). After fertilization, these epigenetic ‘imprints’ are maintained in the somatic lineages and bring about imprinted expression through diverse mechanisms [2,7,8].

Recent studies have shown that oocyte-acquired histone methylation, particularly histone H3 lysine-27 tri-methylation (H3K27me3), can give rise to parentally biased gene expression as well [9,10]. This non-canonical imprinting is limited to the pre-implantation embryo, and is maintained at only a handful of genes in the extra-embryonic lineages [11–16].

Virtually all the ‘classical’ imprinted genes that are controlled by DNA methylation imprints are clustered in large domains. Most of these imprinted chromosomal domains express one or more long non-coding RNAs (lncRNAs), defined as being more than 200 nucleotides in length [17,18]. Accumulating evidence indicates that these lncRNAs contribute to bringing about imprinted gene expression at close by and distant protein-coding genes. Here, we discuss how imprinted non-coding RNAs control gene expression *in cis*, with a particular emphasis on their putative roles in chromatin structure. We also discuss emerging insights into trans-regulatory functions.

## Numerous non-coding RNAs are controlled by genomic imprinting

It is often not well appreciated that numerous non-coding RNAs are imprinted in mammals. For instance, about seven percent of all microRNAs (miRNAs) are imprinted in humans, more than hundred in total. These are mostly transcribed by large host transcription units, each expressing multiple miRNAs [19,20]. One example is the *DLK1-DIO3* imprinted domain on human chromosome 14 (mouse chromosome 12), which expresses 53 miRNAs from a 220 kb polycistronic transcription unit, on the maternal chromosome only. Several of these miRNAs control the levels and/or the translation of mRNAs transcribed by other imprinted genes [21–23]. This highlights the considerable interconnectivity between imprinted loci that has arisen during evolution [24,25]. Another large cluster of imprinted miRNAs maps to human chromosome 19. Interestingly, this ‘C19MC’ cluster is primate-specific and expressed in the placenta predominantly [19,26].

Members of one class of small nucleolar RNAs (snoRNAs) are imprinted as well. These so-called C/D-box snoRNAs are thought to guide 2′-O-methylation on specific RNAs, but their precise roles have remained unclear despite recent functional studies [27–29]. The snoRNA DNA sequences are embedded within large transcription units, similarly as the imprinted miRNAs, each expressing multiple C/D-box snoRNAs [19]. One such a host locus is the imprinted *DLK1-DIO3* domain, which besides many miRNAs, expresses 38 C/D-box snoRNAs from its maternally expressed ncRNA polycistron. The best-studied cluster of imprinted snoRNAs resides within the *SNRPN-UBE3A* imprinted domain, which expresses 81 C/D-box snoRNAs from a large polycistronic gene expressed on the paternal chromosome only [19,30].

With respect to chromatin regulation, the most relevant non-coding RNAs are the lncRNAs [2]. In fact, imprinted lncRNAs were amongst the first discovered long non-coding RNAs and have provided many broadly relevant insights [17]. Most imprinted chromosomal domains express at least one lncRNA and these are RNA Polymerase-II transcribed. The very first example was *H19* at the imprinted *Igf2-H19* domain. This maternally expressed lncRNA was originally described as one of the most highly expressed RNAs during embryonic development, exerting growth-regulating functions [31]. More recent, mechanistic studies revealed that it produces a miRNA (miR-675) that influences muscle development and exerts growth-repressive effects in the placenta [32–34].

Most imprinted lncRNAs originate from their domain's ICR, which acts as a promoter on the unmethylated parental copy. Some are spliced, others not, and several imprinted lncRNAs are retained in the nucleus. These nuclear lncRNAs show different degrees of *cis*-accumulation onto their locus and exert long-range repressive effects, at some loci across several megabases of chromatin [3,17,35].

## Gene regulatory roles of imprinted lncRNAs

In general, lncRNA expression can influence the transcription of protein-coding genes in many different ways [36]. Despite tremendous efforts, however, it has remained complicated to conclude whether observed effects are due to an lncRNA itself or to its transcription [37].

**Figure 1.**
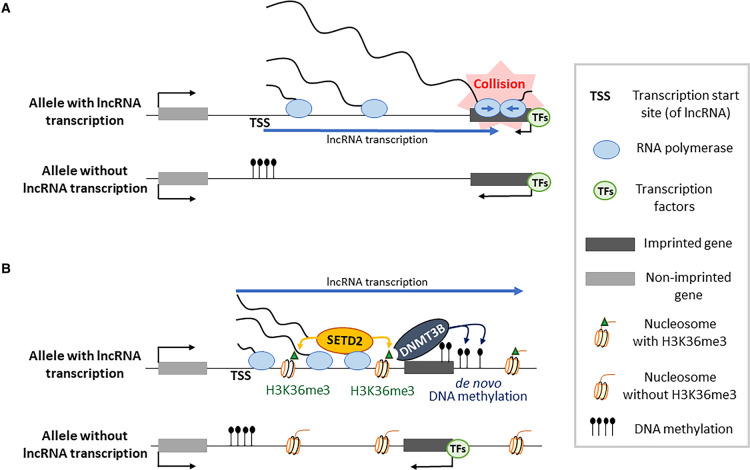
lncRNA-transcription-mediated interference mechanisms. (**A**) One model of transcriptional interference involves collision of RNA Poymerase-II complexes (blue circles). High transcription of an imprinted lncRNA prevents elongation at an overlapping protein-coding gene (black rectangle) transcribed in the opposite direction. In this unidirectional repression model, the promoter of the target gene may show recruitment of transcription factors (TFs, green circles) on both the parental chromosomes. (**B**) Research on several imprinted genes has evoked another unidirectional model, involving promoter occlusion and repression. lncRNA transcription through a protein-coding gene mediates H3K36me3. This involves SETD2, a KMT brought to the chromatin through interaction with RNA-PolII. This induces *de novo* DNA methylation by DNMT3B, a methyltransferase that recognizes H3K36me3 through its PWWP domain. Chromatin associated with the target promoter/CpG island may acquire other covalent histone modifications as well — particularly H3K9me3 — with the combined modifications preventing TF binding.

Extensive research during the last years has evoked different models of how lncRNA transcription could interfere with the expression of close by other genes [36,38–40]. As concerns imprinted lncRNAs one transcription-linked mechanism is interference with an overlapping gene transcribed in the opposite orientation (Figure 1A). A well-studied example of this is the imprinted *Snrpn-Ube3a* domain, where a paternally expressed lncRNA crosses almost one megabase of chromatin, including a distally positioned protein-coding gene called *Ube3a*. Transcriptional stalling caused by the collision of RNA pol-II complexes coming from opposite directions may explain the lack of *Ube3a* expression on the paternal chromosome. lncRNA ablation, or expression of truncated forms of the lncRNA that do not overlap *Ube3A*, cause aberrant activation of this gene on the paternal chromosome [41,42]. Similarly, topoisomerase inhibitors that prevent unwinding of the DNA during transcription —and thereby prevent transcriptional elongation of the lncRNA — reactivate the paternal *Ube3A* gene [43]. Concordantly, antisense oligonucleotides against the long transcript crossing the domain result in the activation of the paternal *UBE3A* gene, and such an approach is currently used in different clinical trials to treat Angelman Syndrome, a neuro-behavioural syndrome caused by loss of *UBE3A* expression [44,45].

A similar model has emerged from detailed studies on the imprinted IGF2-receptor (*Igf2r*) locus on mouse chromosome 17, which expresses a 117 kb non-spliced lncRNA called *Airn* that is transcribed oppositely to the *Igf2r* gene and overlaps its promoter [2]. The allelic lncRNA transcription across the paternal *Igf2r* promoter blocks RNA polymerase II recruitment, initially in the absence of repressive chromatin marks. Although in differentiating cells there is acquisition of DNA methylation and histone H3 lysine-9 trimethylation (H3K9me3), which provide an additional layer of repression, continued *Airn* expression is required to keep the paternal *Igf2r* promoter repressed [46–49].

At other imprinted domains, lncRNA transcription through promoters induces chromatin repression at promoters early in development [17]. At the *Gnas* locus on mouse chromosome 2, for instance, a lncRNA transcription unit called ‘Nesp-antisense’ (*Nespas*) overlaps an oppositely transcribed protein-coding gene called *Nesp* [50] Diverse targeting studies in the mouse, including *Nespas* truncations, led to activation of the normally silent paternal *Nesp* allele. This highlights the importance of transcriptional overlap in the promoter repression (Figure 1B), which involves both histone and DNA methylation [51,52]. In a similar manner, at the imprinted *Zdbf2* locus on mouse chromosome 1, transient transcription during preimplantation development of a lncRNA (called *Liz*) brings about DNA methylation, close to the *Zdbf2* gene [53,54]. As part of the mechanism, RNA polymerase-II could bring the KMT SETD2 to the chromatin, which induces histone H3 lysine-36 tri-methylation (H3K36me3) across the transcribed region. This histone modification is recognized by the DNA methyltransferase DNMT3B (through its PWWP domain) subsequently, which induces *de novo* DNA methylation [55–57].

**Figure 2.**
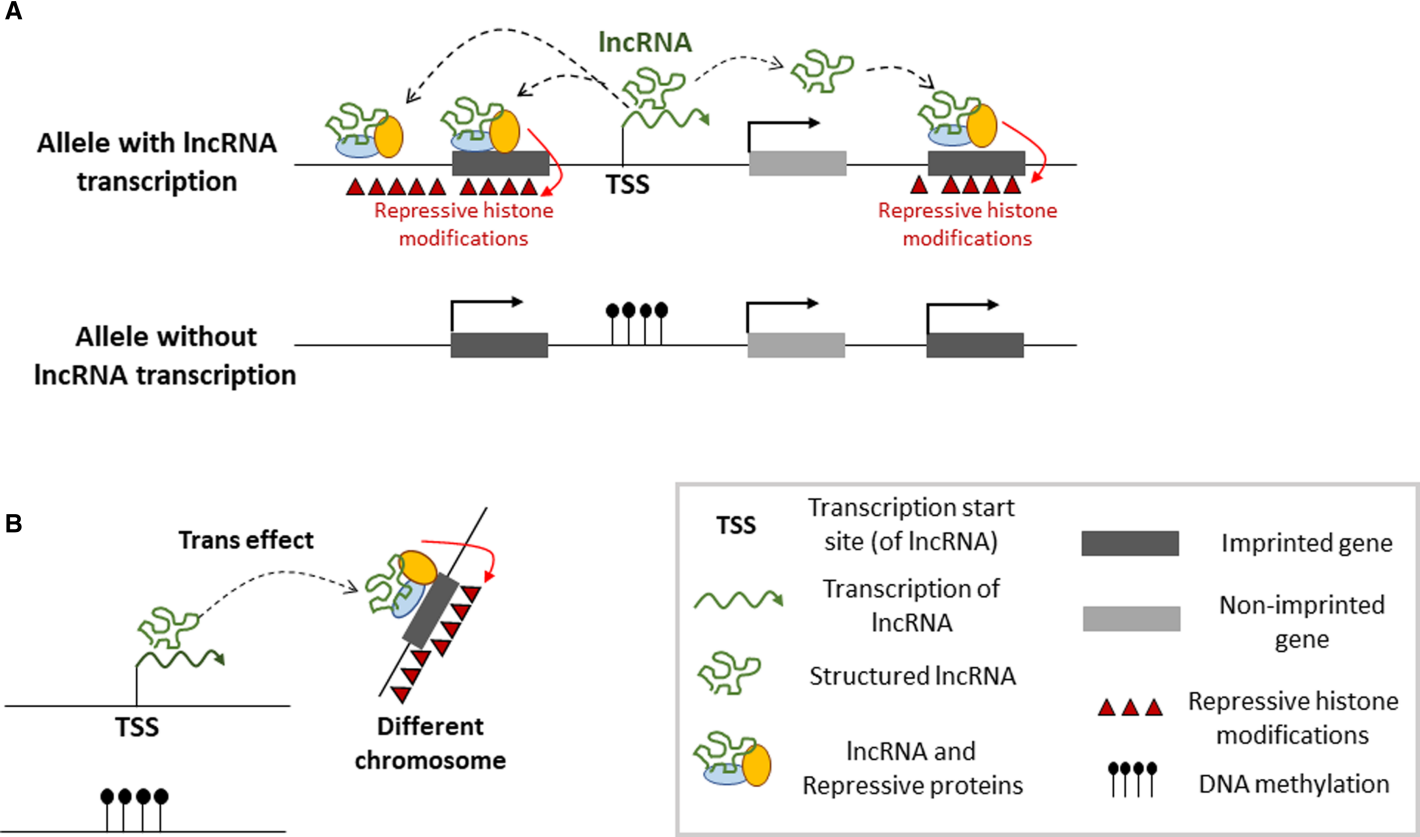
lncRNA-mediated chromatin repression across imprinted gene domains. (**A**) Several imprinted lncRNAs mediate chromatin repression across megabases. This involves enhanced allelic recruitment of PRC1 and -2 complexes, EHMT2 and hnRNPK, in part through direct RNA–protein interactions, leading to allelic spreading of H3K27me3, H2AK119u1 and H3K9me2 across the domain. This bidirectional mechanism of chromatin repression, induced by the lncRNA itself is lineage-specific at several domains and shows certain similarities with X-chromosome inactivation [1]. (**B**) Some imprinted lncRNAs may repress genes on other chromosomes, in *trans*, possibly involving a chromatin-based mechanism that could be similar to *cis* repression [65–68,130].

LncRNA-controlled genes at several imprinted domains are located hundreds to thousands of kilobases away from the lncRNA gene [3,58]. These ‘long-distance’ effects have given rise to models in which the lncRNA itself brings about gene repression (Figure 2). Developmental studies have shown that such long-distance repression occurs in a tissue-specific manner at several of the domains [35]. At the *Igf2r* domain, in the extraembryonic lineages, the paternally expressed *Airn* mediates the allelic repression of several non-overlapping genes positioned up to several megabases away [3,58]. Upon trunctions of this lncRNA, this long-distance repressive effect no longer occurs [46,58,59].

At *Dlk1-Dio3*, similarly, the allelic expression of an lncRNA called *Meg3* is required to repress a distant protein-coding gene involved in Notch signaling, called *Dlk1*, in different somatic tissues [60–62]. Knock-out and overexpression studies have suggested that *Meg3* expression controls genes on other chromosomes as well, including TGF-Β and p53 pathway genes in human cancer cells [63–67]. A similar *trans* effect has been reported for the lncRNA *IPW* generated from the *SNHG14* gene at the *SNRPN-UBE3A* domain (chromosome 15q11–13). This lncRNA dampens *in trans* the promoter of the *MEG3* non-coding polycistron at the *DLK1-DIO3* domain, a process that seems to involve repressive H3K9me3 [68]. This provides yet another example of the intricate regulatory links that exist between imprinted loci [25,69–71].

Another locus showing long-range repressive effects of an lncRNA is the *Kcnq1* domain on mouse chromosome 7. The integrity of a 91 kb lncRNA called *Kcnq1ot1*, particularly a 900 bp region at its 5′ end, is important for the allelic repression of no fewer than eight genes at the proximal and distal parts of this multi-megabase domain [72–76]. Several of the target genes show placental-specific imprinting, indicating that lineage-specific factors likely contribute to the long-range repressive effects of this essential lncRNA [3,58,77]. Combined, the above examples illustrate that several imprinted lncRNAs repress protein-coding genes *in cis*, and that some control genes on other chromosomes as well.

## Imprinted lncRNAs that mediate long-range chromatin repression

Genome-wide reporter-based studies have revealed that many non-imprinted lncRNA genes exert a positive effect on the expression of other genes in their neighborhood [78,79]. These ‘enhancer-like’ effects of lncRNA gene promoters contrast with the observed effects of imprinted lncRNAs, which mostly repress neighboring genes, through nucleation and spreading of repressive histone modifications across large regions [17].

For the imprinted lncRNAs *Kcnq1ot1*, *Airn* and *Meg3* evidence has been obtained for a direct role in chromatin repression. All three are retained in the nucleus and show a certain degree of *cis*-accumulation onto their imprinted domains. This focal accumulation is still detected hours after chemical inhibition of RNA polymerase-II, concordant with the reported intermediate stabilities of these nuclear lncRNAs [58,75,80,81].

Another similarity between *Kcnq1ot1*, *Airn* and *Meg3* is their reported interaction with components of chromatin regulatory complexes (Table 1). In preimplantation embryonic cells and in the placenta, the paternally expressed, 91 kb *Kcnq1ot1* (*Kcnq1* domain) co-localizes and interacts with components of the Polycomb repressive complexes 1 (PRC1) and -2 (PRC2) [74,75,82,83]. It also interacts with EHMT2 (also called G9A) [74], a lysine methyltransferase (KMT) that methylates lysine-9 on histone H3. Concordantly, there is allelic acquisition of EZH2 (PRC2)-mediated H3K27me3, RING1B (PRC1)-mediated H2AK119u1 and EHMT2-mediated H3K9me2 across the paternally repressed genes in trophoblast cells and in the placenta [58,75,77]. In trophoblast stem cells (TSCs) that expressed a truncated form of *Kcnq1ot1*, H3K27me3 levels were strongly reduced across the entire *Kcnq1* imprinted domain [58,74]. *Ehmt2* knock-out in mice gave biallelic expression of several of the placental-specific *Kcnq1ot1* targets in the placenta [84], and, similarly, the essential PRC2 component EED contributes to the process as well [85].

**Table 1:** Chromatin repressive functions of imprinted lncRNAs

lncRNA	Imprinted gene domain	chromatin repressive effect(s) of the lncRNA	References
*Kcnq1ot1* (previously called *Lit1*)	*Kcnq1* domain	* Gene repression *in cis*.	[58,74,75,83,84]
		* Enhances allelic recruitment of PRC1/2, EHMT2 and hnRPNK.	
		* Allelic enrichment of H3K27me3, H2AK119u1 and H3K9me2 across broad regions.	
		* Interacts with CTCF and influences higher-order chromatin features.	
*Airn* (previously called *Air*)	*Igf2r* domain	* Mediates gene repression *in cis*.	[3,46–48,58,59,87]
		* Enhances allelic recruitment of PRC2, EHMT2 and hnRPNK.	
		* Allelic enrichment of H3K27me2, H2AK119ub and H3K9me2 across broad regions.	
		* Interacts with CTCF and influences higher-order chromatin features.	
*Meg3* (also called *Gtl2*)	*Dlk1-Dio3* domain	* Its expression mediates gene silencing *in cis*.	[61,63,65,82,83,131]
		* Likely represses genes *in trans* as well.	
		* Interacts with PRC2 components (EZH2, JARID2) and possibly also with hnRNPK.	
		* Maintains allelic H3K27me3 enrichment at target genes.	
*IPW* (?)	*SNRPN-UBE3A* domain	* Exerts a repressive effect *in trans*.	[68]
		* Influences H3K9me3 levels at its *trans* target (*Meg3* gene).	
*H19* (?)	*Igf2-H19* domain	* Gene repressive effects *in trans*, on other imprinted loci.	[130]
		* Modulates recruitment of the Methyl CpG Binding Domain-1 (MBD1) complex and its associated KMTs.	
		* Modulates H3K9me3 levels at putative target loci.	

A recent study suggests that the *Kcnq1ot1* lncRNA interacts with the nuclear matrix protein hnRNPK. This RNA-interacting protein is essential for the PRC2-mediated H3K27me3 across the imprinted *Kcnq1* domain in TSCs [58]. One emerging model (Figure 2) is that hnRNPK enhances the recruitment and spreading of PRC1 complexes, a process that initiates at CpG islands that were already bound by PRC complexes beforehand [58,86].

A similar picture has emerged for the paternally expressed, 117 kb lncRNA *Airn* (*Igf2r* domain), which in the extraembryonic lineages represses multiple genes across several megabases. In murine TSCs and in placenta, a truncated form of this lncRNA no longer gave gene repression in *cis* [3,58,59]. Recent gene targeting studies in mice show that the long-range repressive effects of *Airn* are not mediated by regulatory sequence elements within the *Airn* lncRNA gene, excluding transcriptional interference mechanisms at the distant non-overlapping genes controlled by *Airn*. Rather, these repressive effects correlate with the broad spreading of PRC2-mediated H3K27me3 and PRC1-mediated H2A-lysine-119 mono-ubiquitination (H2AK119u1) on the paternal chromosome predominantly [58,87]. *Airn* levels are crucial for the allelic recruitment of RING1B (PRC1) and EZH2 (PRC2). Enhancing lncRNA *Airn* copy numbers per cell, by CRISPR-VP16 mediated transcriptional activation, gave enhanced recruitment of PRC complexes onto the paternal chromosome [58]. *Airn* had been shown earlier to facilitate EHMT2 recruitment, which correlates with paternal allele-specific H3K9me2/3 enrichment [59]. Also *Airn* lncRNA seems to interact with hnRNPK and this could enhance recruitment of PRC complexes to the chromatin [88]. In agreement with this hypothesis, the allelic enrichment and spreading of H3K27me3 across the large *Igf2r* domain requires continued expression of the hnRNPK protein in TSCs [58].

*Meg3* lncRNA seems to have a similar mode of action, in somatic tissues. Its expression represses *in cis* a developmental gene called *Dlk1*, located on the proximal side of the imprinted domain [61]. Different studies have reported *Meg3* association with PRC2 components (EZH2 and JARID2) and RNA precipitation assays on cross-linked chromatin suggest binding to hnRNPK as well [58,82,83]. In the absence of *Meg3* lncRNA, there is no longer acquisition of allelic *Dlk1* repression, and this is observed following depletion of EZH2 (PRC2 complex) as well [61]. Similarly as for *Airn* and *Kcnq1ot1* [58], the combined data suggest that *Meg3* lncRNA enhances in an allelic manner the histone modifying activities and possibly also the spreading of PRC complexes, through still poorly understood mechanisms (Figure 2).

The above examples evoke similarities with X-chromosome inactivation in females, which is a *cis* repressive mechanism controlled by an lncRNA (called *Xist*) that involves PRC1, PRC2, EHMT2 and hnRNPK, and other proteins not yet been explored in genomic imprinting [1,88,89]. However, care needs to be taken before drawing firm conclusions. The methodologies used to explore *Xist*, for instance, have been more focused on the lncRNA itself, with functional identification of chromatin-binding RNA motifs. Complementary technologies have also confirmed a direct interaction between *Xist* and hnRNPK, which has not yet been shown for *Airn*, *Kcnq1ot1* or *Meg3* [90–93].

## Emerging roles of imprinted lncRNAs in chromatin architecture

Because of the parental allele-specific DNA methylation imprints, at several imprinted domains there is allelic association of chromatin structural proteins. At several ICRs, and also at secondary DMRs at which the allelic methylation is acquired during development, there is binding of CCCTC-binding factor (CTCF) to the unmethylated allele only (the protein does not bind methylated DNA) [94–97]. This allelic CTCF binding and the CTCF-associated cohesin complexes contribute to imprinted gene expression [98]. Particularly, CTCF mediates long-range chromatin loops with distant other regions on the CTCF bound parental chromosome. Recent studies have explored these structural interactions by using allelic ‘chromosome conformation capture’ (3C) and 3D DNA FISH-based approaches [98].

At the *Igf2-H19* locus, CTCF binding to the unmethylated copy of the ICR brings this region in close proximity to distal regions on the maternal chromosome. Both in mice and humans, this insulates the *Igf2* gene from its distally located enhancers, thus leading to the imprinted *Igf2* expression from the paternal chromosome mostly [96,99–102].

At the *Dlk1-Dio3* domain, CTCF binds the promoter–CpG island of the *Meg3* gene, on its unmethylated maternal copy only [96,103]. Also here, allelic CTCF recruitment brings about specific long-distance structural interactions on the maternal chromosome predominantly. Particularly, the *Dlk1* gene shows close proximity to the lncRNA focus on the maternal chromosome, and this proximity effect contributes to its imprinted expression from the paternal chromosome predominantly [96]. Interestingly, 3D distance measurements between FISH probes show that the imprinted domain is more loosely compacted on the maternal chromosome (compared with the paternal chromosome), which may facilitate the observed CTCF-mediated looping patterns [96,104].

A similar picture has emerged for the *Kcnq1* imprinted domain. Here, CTCF binds the unmethylated paternal copy of the ICR, which also comprises the promoter that drives *Kcnq1ot1* expression on this parental chromosome [105]. The allelic CTCF binding mediates specific long-range interactions on the paternal chromosome, detected by 3C-based technology, that correlate with the allelic expression of several genes within the domain [75,102,106]. Another locus that shows both allelic CTCF binding and allelic lncRNA expression is the imprinted *Zdbf2* domain [107].

Could the allelic lncRNA expression and the allelic binding of CTCF be mechanistically linked? Possibly, transcription factor binding and lncRNA promoter activity keep CTCF binding sites unmethylated, thus ensuring the continued allelic association of this chromatin structural protein (which does not bind methylated DNA [97]). Continued promoter activity at *Meg3* protects indeed against the acquisition of *de novo* DNA methylation in early embryonic cells [108,109]. Point mutations within transcription-factor binding sites at the ICRs of the human *IGF2-H19* and *KCNQ1* domains have provided evidence for such a scenario as well [110–114]. Conversely, CTCF itself may protect the unmethylated allele against *de novo* DNA methylation [110,111,115,116], thus ensuring continued transcription of the lncRNA from the unmethylated parental allele only.

Since *Meg3*, *Kcnq1ot1* and *Airn* show a relatively stable focal accumulation onto their locus [58,61,75,80], this could locally influence CTCF-linked higher-order chromatin structure. CTCF comprises indeed a putative RNA binding domain (RBD) that is functionally important [117,118]. Recent studies suggest that binding of locally transcribed RNAs to the RBD is important for CTCF's association to many of its recognition sites in the genome. This impacts the 3D organization of the genome through the formation of specific chromatin loops [117,118]. It remains to be explored in mice on an F1 background between two phylogenetically distant strains whether there are direct allelic interactions between CTCF and imprinted lncRNAs and to what extent these may influence chromatin loop formation.

How and when lncRNA–protein compartments are formed at imprinted loci, and what controls their developmental regulation, remains unclear. Structural RNA features could be important. Several recent studies explored in detail the structure of *MEG3 in vitro* and in cells [63,119], and interacting RNA loops within the lncRNA were shown to be essential for the *trans* effects of *MEG3* on the p53 pathway in cancer cells [63]. Whilst the RNA sequences of imprinted lncRNAs are generally not well conserved, specific secondary and tertiary structures may be comparable between different mammalian species, and may be important as docking sites for RNA–protein interactions.

Specific RNA sequence elements could be important as well; for instance in the association of lncRNAs to specific target genes in *trans*. In one interesting study on human cancer cells, expression of *MEG3* modulated the expression of TGF-B pathway genes, and this was linked to the formation of RNA–DNA triplex structures across several of these target genes [65]. Although further studies are required, such a process could provide specificity to the *trans* roles of lncRNAs.

The non-imprinted lncRNAs *MALAT1* and *NEAT1* are linked to the formation of membrane-less nuclear bodies called speckles and paraspeckles in specific cell types and under particular conditions [120–122]. Furthermore, emerging evidence on the heterochromatin-linked satellite RNAs and other non-imprinted RNAs suggest that RNA–protein aggregates can potentially form through liquid-liquid phase separation (LLPS) mechanisms (reviewed in [123], an aspect that has not yet been explored in the context of imprinted domains.

**Figure 3.**
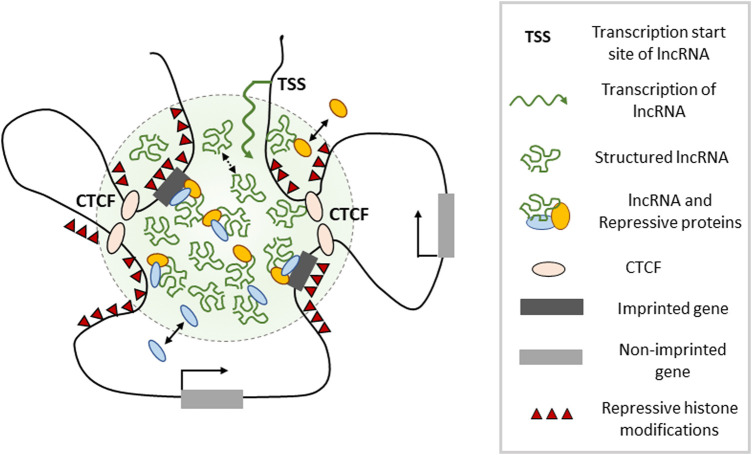
lncRNA–protein aggregates in chromatin architecture and gene expression. The figure depicts a model in which the imprinted lncRNA accumulates in proximity to its transcription site and interacts with different chromatin repressive complexes (PRC1/2, EHMT2, others). This leads to the formation of relatively stable RNA–protein aggregates (green shading), possibly in part through LLPS. Chromosomal gene loci that are in close proximity to the aggregate acquire repressive histone modifications and become silenced. This process could be facilitated by positive effects of the lncRNA on CTCF binding, thereby ensuring appropriate long-range chromatin interactions that bring target gene(s) in close vicinity to the lncRNA–protein aggregate. The model could also explain lncRNA effects in *trans*, on genes located on other chromosomes, in case these are positioned close to the RNA–protein aggregate(s), at least part of the time.

Sub-nuclear localization could impact the process as well, given that at the *Dlk1-Dio3*, *Kcnq1* and other imprinted domains, the lncRNA-expressing parental chromosome displays a more central localization in the nucleus than the opposite parental chromosome [80,124]. The available data so far evoke a model in which focal accumulation of lncRNA and associated chromatin-regulatory complexes creates an aggregate-like organization that brings specific loci in close proximity through protruding chromatin-loop formation and mediates gene repression (Figure 3). At some imprinted domains, interestingly, lncRNA/protein compartments seem to exclude RNA polymerase-II [75], which could be an important aspect of the imprinting process as well. LncRNA-mediated gene repression at imprinted domains is a rather complicated business, and we are only at the beginning of understanding its intricacies.

## Perspectives

- Imprinted gene domains have provided strong paradigms for exploring the regulation and roles of lncRNAs in mammals. Ongoing research efforts unravel *cis*-regulatory chromatin mechanisms and explore how these compare to emerging *trans* roles of imprinted lncRNAs.
- Besides transcriptional interference mechanisms mediated by the expression of lncRNA genes, it is now well accepted that several imprinted lncRNAs themselves control gene repression. These *cis-*repressive actions of lncRNAs likely impact chromatin architecture, involve lncRNA–protein interactions, and specific RNA secondary and tertiary structures could be essential as well. In principle, reported *trans* effects involve the lncRNAs themselves as well [63,65,66,68]. One possibility is that *trans* targets would be transiently positioned in close proximity to lncRNA–protein aggregates, and several recent studies have started to explore this intriguing possibility [102,125].
- Novel CRISPR technologies may help to distinguish between the effects of lncRNA transcription and those of the imprinted lncRNA transcripts *per se* [126,127]. Future research should also unravel which sequence motifs and secondary structures within lncRNAs are important for chromatin repression and architecture, and how these control association of specific lncRNA-interacting proteins. Finally, it is timely to determine the importance of lncRNAs and chromatin architecture in human imprinting disorders (IDs) [4,69]. Initial studies have reported altered chromatin structural interactions within the *KCNQ1* and *IGF2-H19* domains in the growth disorders Beckwith-Wiedemann Syndrome (BWS) and Silver-Russell Syndrome (SRS) [102,128,129].

## Abbreviations

3C: chromosome conformation capture
CTCF: CCCTC-binding factor
DMR: differentially methylated domain
FISH: fluorescence *in situ* hybridization
ICR: imprinting control region
LLPS: liquid-liquid phase separation
MBD: Methyl CpG Binding Domain
PRC: Polycomb Repressive Complex
TAD: topologically associating domain
